# Enhanced Detection of *Mycobacterium bovis*-Specific T Cells in Experimentally-Infected Cattle

**DOI:** 10.3389/fvets.2021.676710

**Published:** 2021-07-14

**Authors:** Paola M. Boggiatto, Carly R. Kanipe, Mitchell V. Palmer

**Affiliations:** ^1^Infectious Bacterial Diseases Research Unit, National Animal Disease Center, Agricultural Research Service, United States Department of Agriculture, Ames, IA, United States; ^2^Immunobiology Program, Iowa State University, Ames, IA, United States; ^3^Oak Ridge Institute for Science and Education (ORISE), Oak Ridge, TN, United States

**Keywords:** Bovine tuberculosis, *Mycobacterium bovis*, T cell responses, proliferation, IFN-g, functional potential

## Abstract

Bovine tuberculosis (bTB), caused by infection with *Mycobacterium bovis*, continues to be a major economic burden associated with production losses and a public health concern due to its zoonotic nature. As with other intracellular pathogens, cell-mediated immunity plays an important role in the control of infection. Characterization of such responses is important for understanding the immune status of the host, and to identify mechanisms of protective immunity or immunopathology. This type of information can be important in the development of vaccination strategies, diagnostic assays, and in predicting protection or disease progression. However, the frequency of circulating *M. bovis*-specific T cells are often low, making the analysis of such responses difficult. As previously demonstrated in a different cattle infection model, antigenic expansion allows us to increase the frequency of antigen-specific T cells. Moreover, the concurrent assessment of cytokine production and proliferation provides a deeper understanding of the functional nature of these cells. The work presented here, analyzes the T cell response following experimental *M. bovis* infection in cattle via *in vitro* antigenic expansion and re-stimulation to characterize antigen-specific CD4, CD8, and γδ T cells and their functional phenotype, shedding light on the variable functional ability of these cells. Data gathered from these studies can help us better understand the cellular response to *M. bovis* infection and develop improved vaccines and diagnostic tools.

## Introduction

Bovine tuberculosis (bTB), is a chronic bacterial infection caused primarily by *Mycobacterium bovis*, a member of the *Mycobacterium tuberculosis* complex (1). This group of genetically-related mycobacteria also includes *M. tuberculosis, M. cannetii, M. africanum, M. pinnipeii, M. microti, M. caprae*, and *M. mungi*, which are known to infect and result in similar disease pathology in multiple hosts (2). Worldwide, bTB is a major cause of economic hardship. In 1995, it was estimated that bTB causes >50 million cattle infections resulting in $3 billion of losses annually (3, 4). The number of infected cattle and associated economic loss are likely higher today and its zoonotic nature poses a legitimate public health risk.

As with other intracellular pathogens, protective immune responses against tuberculosis (TB) are associated with interferon gamma (IFN-γ) production derived from T helper 1 (T_H_1) CD4 T cells (5–9). Delayed-type hypersensitivity reactions and IFN-γ release assays (IGRA) are commonly used to assess Mycobacterial reactivity or infection in various species [reviewed in Schiller et al. (10), Walzl et al. (11)]. However, these responses may not necessarily correlate with protection. In cattle, vaccination against bTB results in the induction of IFN-γ responses that can be measured *ex vivo* following overnight antigen stimulation, yet neither the presence nor the levels of IFN-γ induced translate into levels of protection afforded by vaccination (4, 11, 12).

Immune protection from future infections is mediated by the induction and maintenance of memory responses. Memory T cells are a heterogenous population of cells including T effector memory (T_EM_), T central memory (T_CM_), and T resident memory (T_RM_) cells, which display distinct functional, effector, and migratory phenotypes (13). T_EM_ tend to have a fast response to antigen, retain their effector function (i.e., cytokine production), and are relatively short-lived. In contrast, T_CM_ respond slower to antigen, show increased proliferative capabilities, can generate T effector and T_EM_ cells, and are long-lived. Previously, our laboratory demonstrated that following *M. bovis* infection in cattle, both T_EM_ and T_CM_ CD4 T cells are generated (14). In addition, we demonstrated that following *M. bovis* infection, bovine T_CM_ are highly proliferative to antigen stimulation, and that T_CM_ cells can revert in phenotype to generate T_EM_ and T effector phenotypes (14).

While IFN-γ may not serve as a correlate of protection, it nevertheless plays a central role in the response to TB. Long-term culture systems, that measure IFN-γ from T_CM_ cells, appear to be better predictors of vaccine efficacy as compared to *ex vivo* IFN-γ production (15–17). These data would suggest that perhaps the T cell source of IFN-γ is a better predictor of protection rather than the overall levels of IFN-γ. Since proliferation is another characteristic feature of memory responses, typically associated with T_CM_, we wondered if the concurrent assessment of proliferation and IFN-γ production would allow us to better understand the source of IFN-γ and the overall functional phenotype of memory responses following *M. bovis* infection.

In the work presented here, we utilize an *in vitro* recall response assay, whereby antigen-specific cells are expanded, and proliferation and IFN-γ production are assessed concurrently. Additionally, we assess the potential of these antigen-specific cells to produce IFN-γ via restimulation, thereby enhancing their detection.

## Materials and Methods

### Animals and *Mycobacterium bovis* Aerosol Challenge

Holstein steers (~6 months of age) were obtained from a tuberculosis-free source and housed at the National Animal Disease Center, agricultural biosafety level 3 (AgBSL3) animal facility. Animals were allowed to acclimate for 2 weeks prior to challenge. All animal studies were conducted with approval from the Institutional Animal Care and Use Committee (AICUC) at the National Animal Disease Center in Ames, Iowa.

*Mycobacterium bovis* strain 10-7428, a field strain of low passage (<3), which has been shown to be virulent in a calf aerosol model (18). The inoculum was prepared using standard techniques (19) in Middlebrook's 7H9 liquid media (Becton Dickinson, Franklin Lakes, NJ) supplemented with 10% oleic acid-albumin-dextrose complex (OADC) (Difco, Detroit, MI) plus 0.05% Tween 80 (Sigma Chemical Co., St. Louis, MO). Mid log-phase growth bacilli were pelleted by centrifugation at 750x g, washed twice with phosphate buffered saline (PBS) (0.01 M, pH 7.2) and stored at −80° C until used. Frozen stock was warmed to room temperature and diluted to the appropriate cell density in 2 ml of PBS. Bacilli were enumerated by serial dilution plate counting on Middlebrook's 7H11 selective media (Becton Dickinson). A single dose was determined to be 1.12 × 10^4^ CFU per steer.

*M. bovis* aerosol infection in cattle has been previously described (18, 20, 21). Briefly, eight (8) steers were infected with a single dose of virulent *M. bovis* strain 10-7428 by nebulization of inoculum into a mask (Equine AeroMask®, Trudell Medical International, London, ON, Canada) covering the nostrils and mouth. Six (6) age-matched steers were used as non-infected controls. All experimental animal procedures were conducted in accordance with recommendations in the Care and Use of Laboratory Animals of the National Institutes of Health and the Guide for the Care and Use of Agricultural Animals in Research and Teaching (22, 23). All animal-related procedures were also approved by the USDA-National Animal Disease Center Animal Care and Use Committee.

### Isolation of Peripheral Blood Mononuclear Cells

Whole blood was collected via venipuncture of the jugular vein into EDTA tubes. Blood was processed for isolation of PBMC as described earlier (24), with some modifications. Briefly, 10 ml of blood were diluted 1:2 in sterile, culture grade, Dubelcco's phosphate-buffered saline (DPBS) (Gibco, Thermo Fisher, Waltham, MA) and centrifuged at 1,200 × g for 30 min at room temperature (RT). The buffy coats were harvested and overlayed onto 5 ml of 1.077 Ficoll (Sigma-Aldrich, St. Louis, MO), and centrifuged again at 1,200 × g for 30 min at RT. PBMC were then harvested and washed once in sterile PBS at 300 × g for 10 min. Cells were then counted on a hemocytometer using trypan blue staining to determine number and viability. Cells were then resuspended to the desired concentration using complete RPMI 1640 (Gibco Life Tech, Thermo Fisher) media, as described previously (24).

### PBMC Labeling, *in vitro* Antigen Stimulation, and Restimulation

In order to assess antigen-specific responses, PBMC were first labeled using the CellTrace® violet proliferation kit (Invitrogen, Thermo Fisher), according to manufacturer's recommendations. Following labeling, 1 × 10^6^ cells were plated onto 96-well, flat bottom plates and left unstimulated, or stimulated with PPDb (5 μg/well), or Concanavalin A (ConA, 0.5μg/well, Sigma-Aldrich), in a total volume of 200 μl. Cells were then incubated at 37°C with 5% CO_2_ for 7 days. In order to assess intracellular cytokine production, cultured PBMC were treated with either a 1 × solution of eBioscience™ Protein transport inhibitor (500 × Brefeldin A, Thermo Fisher) or with a 1 × solution of eBioscience™ Cell Stimulation cocktail plus Protein transport inhibitor [500 × phorbol 12-myristate 13-acetate (PMA), ionomycin, Brefeldin A] (Thermo Fisher) and incubated overnight for ~16 h prior to harvest on day 7 (24).

### Surface and Intracellular Staining

For surface and intracellular cytokine staining, PBMC were harvested on day 7 of culture and washed with DPBS via centrifugation at 300 × g for 5 min at RT. Staining was performed as described previously (24). Briefly, cells were incubated with a fixable viability dye (eBioscience™, Thermo Fisher) and then stained for surface markers using FITC-labeled anti-bovine CD4 (CC8, Bio-Rad, Hercules, CA), APC-labeled anti-bovine CD8 (CC63, Bio-Rad), and anti-bovine γδ (IgG2b, TCR1-N24, Washington State University, Pullman, WA; BUV-labeled anti-IgG2b, BD Bioscience, San Jose, CA) antibodies. Cells were then fixed and permeabilized using BD Cytofix/Cytoperm™ kit (BD Bioscience) according to manufacturer's recommendation and stained with an anti-bovine, PE-labeled IFN-γ antibody (CC302, Bio-Rad). Cells were resuspended in FACS buffer and then analyzed using a BD FACSymphony™ A5 flow cytometer (BD Bioscience). Data was analyzed using FlowJo® software (Tree Star, Inc., San Diego, CA).

### Statistical Analysis

Statistical analyses were performed using GraphPad Prism 8 (GraphPad software, San Diego, CA). Pair-wise comparisons of means were performed using *t-*tests, and multiple comparisons mixed-effects analysis were performed using Sidak's multiple comparisons test. *P* ≤ 0.05 were considered statistically-significant.

## Results

### T Cell Population Subsets

**Figure 1:**
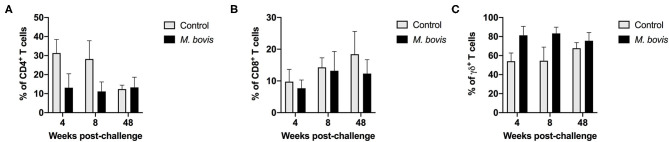
Frequency of T cell subsets in control and *M. bovis* infected animals following challenge and *in vitro* antigen stimulation. PBMC from control (gray bars) and *M. bovis*-infected (black bars) animals were isolated at different time points after infection and stimulated *in vitro* with PPDb. Shown are the frequency of CD4 **(A)**, CD8 **(B)**, and γδ **(C)** T cells following *in vitro* stimulation. Presented are mean frequency values ± S.D.

Analysis of T cell population subsets from cultured PMBC from control and *M. bovis*-infected animals at various time points post-challenge was performed; gating scheme for the analysis is shown in Supplementary Figure 1. Frequencies of CD4, CD8, and γδ T cells did not differ significantly between control and infected animals at all time points analyzed (Figures 1A–C). Overall, frequencies of all three subsets were relatively stable through the course of infection, with γδ T cells comprising the majority of the circulating pool of T cells (Figure 1C).

### T Cell Proliferative Responses Following *M. bovis* Infection

**Figure 2:**
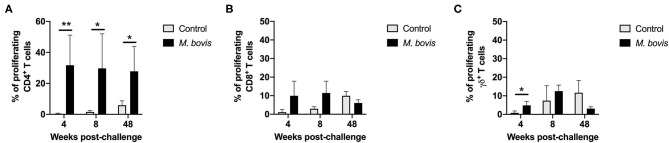
Proliferation responses of T cell subsets from control and *M. bovis*-infected animals, following challenge and *in vitro* antigen stimulation. Shown are the frequencies of proliferating CD4 **(A)**, CD8 **(B)**, and γδ **(C)** T cells for control (gray bars) and *M. bovis* infected (black bars) animals at 4, 8, and 48 weeks post-infection in response to PPDb *in vitro* stimulation. Presented are mean frequency values ± S.D. Statistically significant differences are indicated; *p ≤ 0.05 and **p ≤ 0.01.

In order to assess antigen-specific responses, PBMC from control and *M. bovis*-infected animals were stimulated *in vitro* with PPDb and the frequency of proliferating CD4, CD8, and γδ T cells were determined. Representative histograms for assessment of proliferation can be seen in Supplementary Figure 2. Following antigen stimulation, we observed a significant increase in the frequency of proliferating CD4 T cells from *M. bovis*-infected animals as compared to controls at 4, 8-, and 48-weeks post-infection (Figure 2A). CD8 T cells from infected animals at 4- and 8-weeks post-infection showed an increase in the frequency of proliferating cells, as compared to control animals, however, this change was not statistically significant (Figure 2B). Similarly, proliferation was observed in γδ T cells from *M. bovis*-infected animals, however, the frequency of proliferating cells was only statistically different from control animals at 4 weeks post-infection (Figure 2C). Altogether, these data indicate that the majority of the proliferative response to PPDb antigens occurred within the CD4 T cell compartment of PBMCs from *M. bovis*-infected animals.

### T Cell IFN-γ Responses Following *M. bovis* Infection

**Figure 3:**
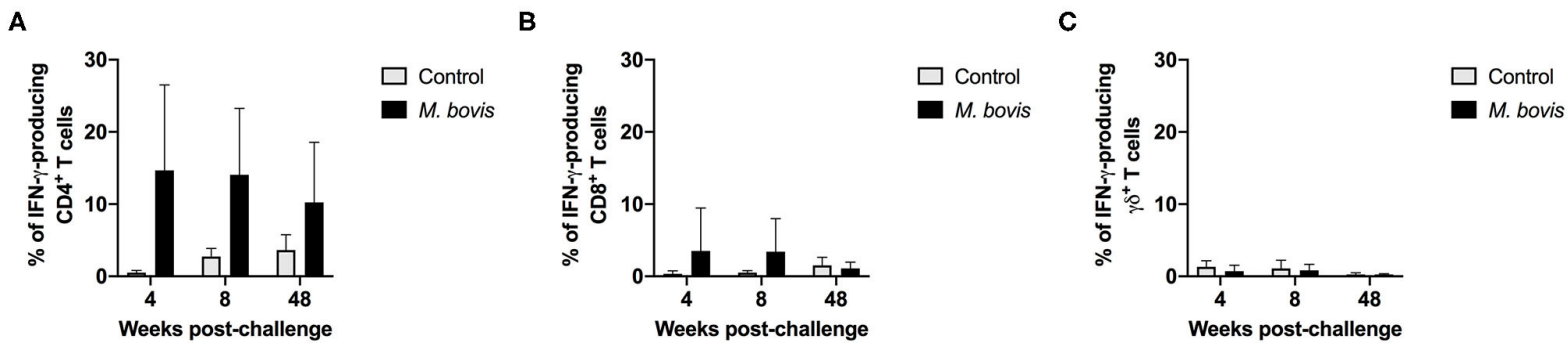
Frequency of IFN-γ responses of T cell subsets from control and *M. bovis*-infected animals following *in vitro* antigen stimulation. Shown are the frequencies of IFN-γ-producing CD4 **(A)**, CD8 **(B)**, and γδ **(C)** T cells from control (gray bars) and *M. bovis-*infected (black bars) animals at 4-, 8-, and 48-weeks post-infection, following *in vitro* PPDb stimulation. Presented are mean frequency values ± S.D.

The frequency of IFN-γ-producing cells following *in vitro* stimulation of PBMC from control and infected animals was also assessed. Similar to the proliferation data, IFN-γ production was predominantly observed within the CD4 T cell compartment of infected animals, at all time points analyzed (Figure 3). However, despite observing an increased frequency of IFN-γ-producing CD4 T cells from *M. bovis*-infected animals as compared to controls, these differences were not statistically significant (*p* = 0.09, 0.08, and 0.30, respectively, for each time point) (Figure 3A). At 4- and 8-weeks post-infection, we observed an increase in the frequency of IFN-γ-producing CD8 T cells as compared to controls, however, this increase was not statistically significant (Figure 3B). The IFN-γ contribution from γδ T cells was relatively minimal, and no differences were seen between infected and control animals (Figure 3C). Congruent with the proliferation findings from above, the majority of IFN-γ produced in response to PPDb antigens is derived from CD4 T cells following *M. bovis* infection.

### Concurrent Assessment of Proliferation and IFN-γ Responses to Mycobacterial Antigens

**Figure 4:**
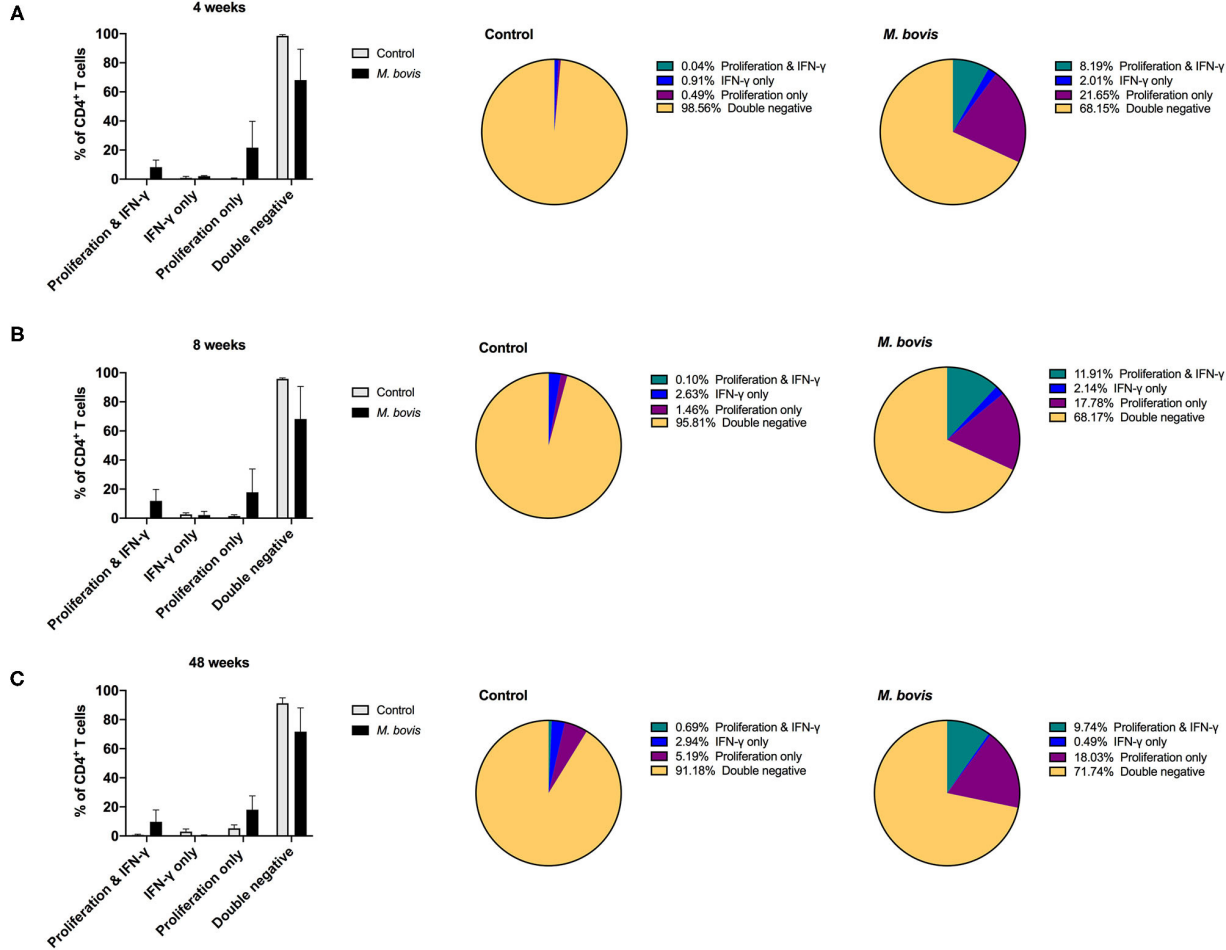
Distinct functional subsets of *M. bovis*-specific CD4 T cells following concurrent assessment of proliferation and IFN-γ production. Bar graphs (left) and pie charts (right) showing the frequency of CD4 T cells with distinct functional phenotypes from control (gray bars) and *M. bovis*-infected animals (black bars) at 4-**(A)**, 8-**(B)**, and 48-**(C)** weeks post-infection. Functional phenotypes are denoted as CD4 T cells that in response to *in vitro* PPDb stimulation show proliferation and IFN-γ production (green), IFN-γ production only (blue), proliferation only (purple), or do not respond [double negative (gold)]. Shown are mean frequencies ± S.D.

Assessing proliferation and cytokine production concurrently, allows for further characterization of the functional potential of antigen-specific cells. By analyzing cells in this fashion, four distinct functional subsets were identified: cells that proliferate and produce IFN-γ (double function), cells that only produce IFN-γ, cells that only proliferate, and cells that neither proliferate nor produce IFN-γ (double negative) in response to antigen (Supplementary Figure 3). These functional subsets were identifiable for CD4 (Figure 4), CD8 (Supplementary Figure 4), and γδ (Supplementary Figure 5) T cells. However, not unexpectedly, these subsets were more discernable within the CD4 T cell compartment, as this is the T cell population with the greatest frequency of cells responding to antigen stimulation. Functionally, antigen-specific CD4 T cells are primarily capable of either proliferating or both proliferating and producing IFN-γ. At 4, 8-, and 48-weeks post-infection, CD4 T cells that proliferate and produce IFN-γ to antigen stimulation comprise approximately 8.19, 11.91, and 9.74% of the total CD4 response, respectively (Figure 4, pie charts, green color). In comparison, cells that only proliferate in response to antigen stimulation make up 21.65, 17.78, and 18.03% at 4, 8-, and 48-weeks post-infection, respectively (Figure 4, pie charts, purple color). These data indicate that a smaller frequency of CD4 T cells display dual function at all times points analyzed. In fact, further analysis of the CD4 antigen-specific response, which excludes the double negative population (i.e., cells not responding to antigen stimulation), revealed that the proliferation-only population comprised over 50% of the response at all time points analyzed (Supplementary Figure 6, top panel). CD4 T cells that showed dual function (i.e., proliferation and IFN-γ) comprised 25–37% of the antigen specific cells (Supplementary Figure 6, top panel, green), while single IFN-γ producers only made up a very small percentage of the population (1–6%) (Supplementary Figure 6, top panel, blue).

Despite the reduced frequency of responding CD8 T cells, a similar pattern of functional phenotype was observed for this subset (Supplementary Figure 4). Proliferative responses made up the majority of the functional potential of CD8 T cells, followed by double producers, and a smaller percentage of IFN-γ-only producing cells (Supplementary Figures 4, 6, middle panel). Interestingly, γδ T cells showed primarily a proliferative response (Supplementary Figure 5), making up over 90% of the antigen-specific response at all time points analyzed (Supplementary Figure 6, bottom panel). Altogether, these data showed that when functional phenotypes are assessed concurrently, proliferation appears to predominate for CD4, CD8, and γδ T cells. Additionally, CD4-, CD8-, and γδ-derived IFN-γ arises from cells that have also proliferated in response to antigen, with a much smaller contribution arising from non-proliferating cells.

### Enhancing Cytokine Production Following Mycobacterial Antigen Stimulation

**Figure 5:**
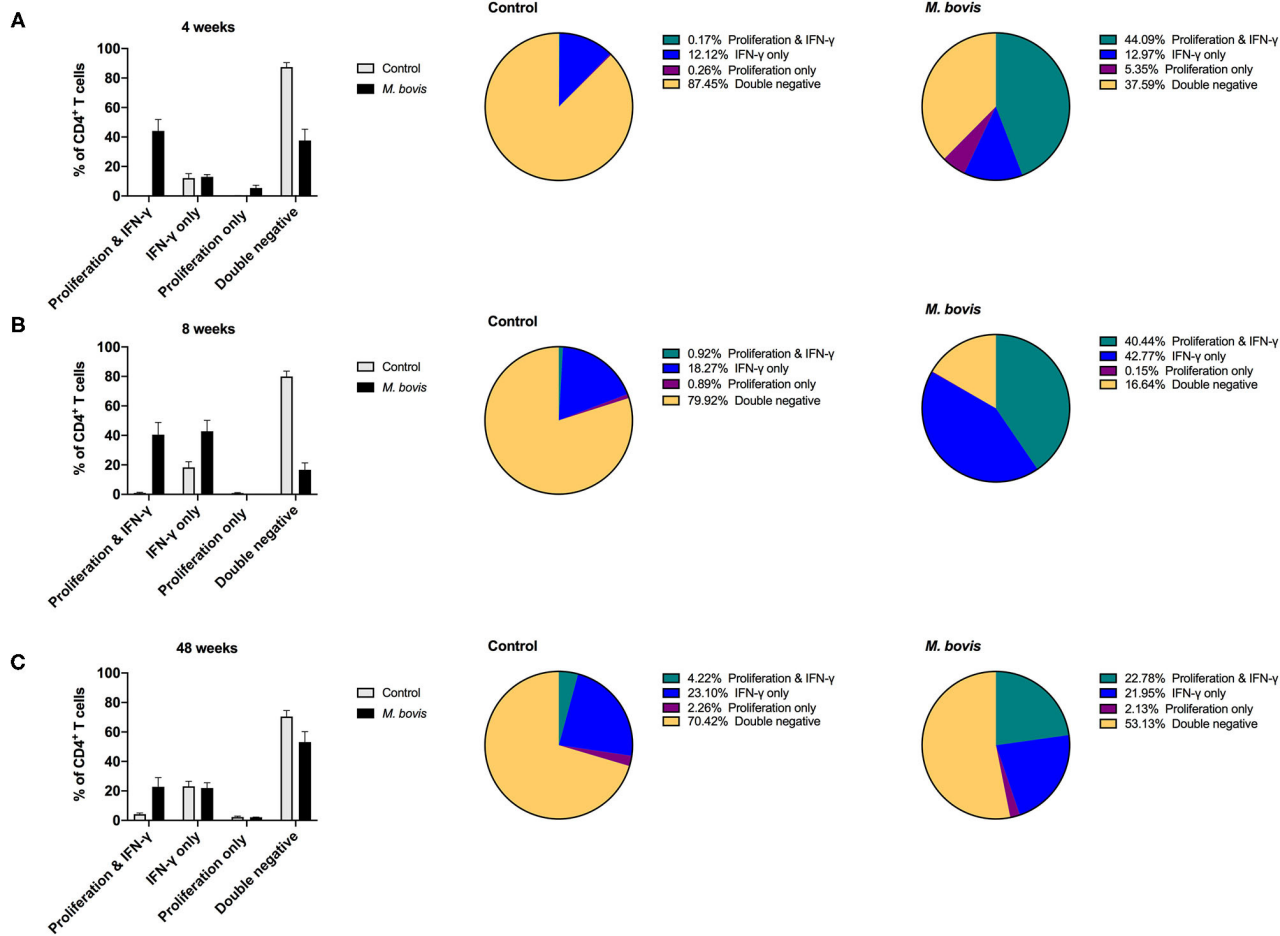
Distinct functional subsets of *M. bovis*-specific CD4 T cells following concurrent assessment of proliferation and IFN-γ production following antigen stimulation and restimulation *in vitro*. Bar graphs (left) and pie charts (right) showing the frequency of CD4 T cells with distinct functional phenotypes from control (gray bars) and *M. bovis*-infected animals (black bars) at 4-**(A)**, 8-**(B)**, and 48-**(C)** weeks post-infection. PBMC were stimulated with PPDb for 7 days and restimulated with PMA/ionomycin overnight for the last 16 h of culture. Proliferation and IFN-γ production were then assessed concurrently via flow cytometry. Functional phenotypes are denoted as CD4 T cells that show proliferation and IFN-γ production (green), IFN-γ production only (blue), proliferation only (purple), or do not respond [double negative (gold)]. Shown are mean frequencies ± S.D.

Above findings indicated that proliferation comprises the majority of the antigen-specific response, and that only a subset of proliferating cells produced IFN-γ in response to PPDb stimulation. We wondered if the remainder of proliferating cells had the potential to make cytokines, when re-stimulation was provided *in vitro*. In order to determine if these cells had the potential to produce IFN-γ, 16 h prior to harvest on day 7, cells were re-stimulated with PMA/Ionomycin, a pan-T cell stimulator, in the presence of brefeldin A, as described previously (24). Indeed, following restimulation with PMA/Ionomycin, we observed a lower percentage of cells that only proliferated (Supplementary Figure 3, last column). This is particularly clear for CD4 T cells from *M. bovis*-infected animals, where cells displaying dual function (proliferation and IFN-γ) now constitute ~44, 40, and 22% of the response at 4-, 8-, and 48-weeks post-infection, respectively (Figure 5). The frequency of proliferating-only CD4 T cells constitutes a mere 5, 0.15, and 2.13% of responding cells (Figure 5).

This switch in the functional profile of antigen-specific T cells following restimulation is also observed in antigen-specific CD8 (Supplementary Figure 7) and γδ (Supplementary Figure 8) T cells, albeit not to the extent that was observed for CD4 T cells. When analyzing this response solely within the subset of cells responding to antigen, CD4 T cells that proliferate and produce IFN-γ constitute 70% of the response at 4 weeks post-infection and 48% at 8- and 48-weeks post infection (Supplementary Figure 9, top row). In comparison, antigen-responsive cells CD8 T cells that proliferate and produce IFN-γ consistently constitute ~30% of the response at 4-, 8-, and 48-weeks post infection (Supplementary Figure 9, middle row, green). At 4- and 48-weeks post-infection, there remains a substantial population of CD8 T cells (33 and 23%, respectively), that only demonstrate proliferative potential despite the addition of restimulation (Supplementary Figure 9, middle row, purple). A similar pattern of response is observed for γδ T cells responding to antigen stimulation; there is an increase in the frequency of proliferating and IFN-γ-producing cells at week 8 post-infection, yet at 4- and 48 weeks post infection proliferation predominates as the antigen-specific response (Supplementary Figure 9, bottom row, green and purple). Altogether, these data demonstrate that *M. bovis*-specific T cells that proliferate to antigen stimulation are all capable of producing IFN-γ, and that this response can be enhanced by providing restimulation.

## Conclusions

Understanding the functional role of T cells following *M. bovis* infection in cattle will provide insights for the development of vaccine and diagnostic interventions. Proliferation and cytokine production are two major functional characteristics of activated, antigen-specific T cells. Production of IFN-γ from T cells is intimately tied with T_H_1 responses, which are necessary for the clearance of intracellular pathogens, including mycobacteria. However, IFN-γ levels do not always correlate with protection (4, 11). In the work presented here, we sought to further dissect the functional potential of *M. bovis*-specific T cells by concurrently assessing two commonly-measured functional phenotypes: proliferation and IFN-γ production. In doing so, we were able to characterize three distinct functional subsets for all three T cell populations: cells with the potential to proliferate and produce IFN-γ (dual function), cells that only produce IFN-γ, and cells that only proliferate in response to antigen stimulation. Furthermore, we demonstrate that T cells with dual function and cells that proliferate-only make up the majority of the *M. bovis*-specific T cell response. We also demonstrate that when restimulated, antigen-specific cells that proliferate-only, are capable of producing IFN-γ.

The frequency of CD4, CD8, and γδ T cells remains relatively stable throughout the course of infection, or at least through the time points analyzed in this study (4, 8, and 48 weeks post-infection). In addition, we found that *M. bovis*-specific proliferative responses can be seen in all three T cell populations, yet significant increases in the frequency of proliferating cells were only observed for CD4 T cells at all three time points. Similarly, IFN-γ responses were primarily seen within CD4 T cells, and to a lesser extent with CD8 T cells. Interestingly, despite this increase in the frequency of IFN-γ-producing CD4 T cells in *M. bovis*-infected animals, these changes were not statistically significant when compared to CD4 T cells from control animals. We attribute this to the variability in responses observed for IFN-γ in outbred populations, such as cattle. It should be noted that moderate variability was also observed for proliferative responses. Cattle represent an outbred population, and while experimental infections provide some level of control, variability in animal responses are expected. Overall, however, we show that proliferation and IFN-γ responses following *M. bovis* infection are primarily found within the CD4 T cell compartment, with CD8 and γδ T cells providing minor contribution to these responses, consistent with previous observations from our laboratory (14).

Concurrent measurement of proliferation and IFN-γ provides another level of insight into the functional potential of antigen-specific T cells. As a result, we were able to identify three distinct antigen-specific T cell populations with functional potential. We observed that following *M. bovis* challenge, there are two proliferating populations, one that produces IFN-γ and one that only proliferates. These two populations could represent distinct effector or memory subsets. We have previously demonstrated that following *M. bovis* infection, cattle do develop T central memory (T_CM_) and T effector memory (T_EM_) responses (14). Furthermore, we have demonstrated that antigen stimulation not only results in proliferation of T_CM_, but also in a switch from T_CM_ to T_EM_, which are capable of producing IFN-γ (14). By measuring the functional phenotypes concurrently, we are likely seeing those distinct subsets. The work presented here did not include surface markers to corroborate memory phenotypes and only characterized proliferation and IFN-γ production. Despite this, it should be noted that by measuring proliferation and cytokine production concurrently, we were able to identify two functionally-distinct populations of cells responding to antigen stimulation. This approach may allow further characterization into other memory and/or effector subsets, which cannot be identified using surface markers as they have either not yet been identified or are not available for cattle.

Concomitant immunity, is a mechanism of immunity whereby a persistent, low-grade infection results in protection from subsequent re-infection with the same pathogen. This type of protection has been shown in other infectious models such as *Leishmania* (25). Unlike the classical idea of “T cell memory,” concomitant immunity is primarily driven by a unique subset of CD4 effector cells that are derived from T_CM_ cells, are non-proliferative, are high IFN-γ producers, and are long-lived but only under conditions of persistent antigen availability [reviewed in Reyed and Rafati (26)]. In the mouse, this subset of T effector cells can be characterized by expression of the surface marker Ly6C (26), but this marker has not been characterized in other species. Therefore, tracking of this specific cell type becomes difficult in other species. This phenomenon, and its potential role in mediating protection in tuberculosis, have been recently described in a non-human primate model (27). Further support for concomitant immunity is found in human cohort studies as well as epidemiological data suggesting that prior infection with *M. tuberculosis* provides protection against subsequent infections (28, 29). The role of concomitant immunity has not yet been explored for bTB. While we may be unable to characterize Ly6C-expressing T effector memory subset based on surface expression markers, it may be possible to characterize these cells based on their non-proliferative and high-IFN-γ expression profile, which this assay would facilitate. Additional surface and intracellular markers could be added in order to expand the profiling of memory subsets using this assay. We propose that by refining the concurrent analysis of functional phenotypes, we may be able to explore some of these questions in cattle, thus providing further insights into the cellular immune response to *M. bovis* in its natural host.

The presence of antigen-specific cells that proliferate but do not produce IFN-γ in response to antigen stimulation led us to ask the question about the functional potential of these cells. To address this, we stimulated these cells with PMA/Ionomycin and measured cytokine production. Interestingly, as seen with T cells from cattle vaccinated against brucellosis (24), this subset of proliferating T cells is capable of producing IFN-γ. These data beg the question as to why the distinction in functional phenotype. As mentioned earlier this could be related to memory subtypes (T_CM_ vs. T_EM_), or perhaps this distinction is related to the nature of antigen stimulation. We assume that these antigen-specific T cells constitute a heterogenous populations of cells with a wide T cell repertoire and antigenic specificity. One hypothesis would be that the quantity and quality of the antigen may be responsible for driving function. Indeed, it has been previously shown that thresholds of T cell receptor signaling and duration of signaling determine T cell fate including functions such as cytokine production and proliferation [reviewed in Zikherman and Au-Yeung (30)]. We cannot discard the possibility that the antigen in this assay reaches varying degrees of stimulation for different T cell receptors. Further analysis utilizing defined antigens (i.e., single peptides or peptide cocktails) for stimulation using this assay, would allow us to determine if antigen-specificity and/or availability drives the functional distinction observed here.

Restimulation with PMA/ionomycin also allowed for the enhanced detection of antigen-specific, IFN-γ-producing CD4 cells (i.e., cells proliferating and producing IFN-γ). The restimulation step demonstrated that these proliferating cells are capable of producing IFN-γ when added stimulation is provided. However, we did not assess whether these cells could produce any other cytokines. Polyfunctional T cells (i.e., cells with the ability to produce multiple cytokines) have been shown to correlate with protection in various infectious models (31–33). However, the role of polyfunctional T cells in TB remains poorly understood, with conflicting data for their role in protection vs. active disease (34, 35). Work from our laboratory has shown that in cattle polyfunctional (IFN-γ/TNF-α/IL-2) CD4 T_CM_ cells are associated with protective responses following BCG vaccination, while IFN-γ/TNF-α T_CM_ cells are associated with higher bacterial burdens (36). The data presented here suggests that a large proportion of *M. bovis*-specific CD4 T cells only proliferate upon antigen stimulation, but are capable of producing cytokines in response to restimulation. It may be possible that a significant portion of antigen-specific cells are missed when cytokine analysis is performed in isolation. Assessment of cytokine polyfunctionality using restimulation, as described here, may provide a way to enhance our ability to detect these T cell subsets with polyfunctional phenotypes.

Altogether, the work presented here utilized an *in vitro* assay relying on enrichment of *M. bovis-*specific T cells via antigen stimulation to characterize functional phenotypes of proliferation and/or IFN-γ-production. The data demonstrate that the majority of antigen-responsive CD4 T cells proliferate in response to antigen, followed by cells capable of proliferating and producing cytokine. This type of approach, concurrent assessment of two major functions of activated T cells, along with further phenotypic analysis, are likely to increase our fundamental understanding of T cell responses involved in bTB.

## Data Availability Statement

The original contributions presented in the study are included in the article/Supplementary Material, further inquiries can be directed to the corresponding author/s.

## Ethics Statement

The animal study was reviewed and approved by National Animal Disease Center Institutional Animal Care and Use Committee (NADC IACUC).

## Author Contributions

PB, CK, and MP: experiment design, sample collection, experiments, and manuscript editing. PB: data analysis and manuscript preparation. All authors contributed to the article and approved the submitted version.

## Conflict of Interest

The authors declare that the research was conducted in the absence of any commercial or financial relationships that could be construed as a potential conflict of interest.

## Supplementary Material

Supplementary Figure 1 Flow cytometry gating scheme for T cell subsets. Shown are representative dot plots demonstrating gating strategies for lymphocytes and singlet discrimination based on FSC and SSC. Also shown are live/dead discrimination based on uptake of a fixable viability dye, and CD4, CD8, and γδ gating based on SSC and fluorescent signal.

Supplementary Figure 2 Flow cytometry analysis of proliferating cells based on CellTrace™ violet dilution. Shown are representative histograms for determination of the frequency of proliferating CD4, CD8, and γδ T cells without stimulation (first column), PPD-B antigen stimulation (middle column), and ConA stimulation (last column).

Supplementary Figure 3 Flow cytometry gating for the concurrent assessment of proliferation and IFN-γ responses for T cell subsets. Shown are representative dot plots for cells gated on CD4 (top row), CD8 (middle row), and γδ (bottom row) following *in vitro* culture without stimulation (first column), with PPDb stimulation (middle column) and PPDb + PMA/Ionomycin (last column), for the assessment of proliferation (CellTrace dilution, y-axis), and IFN-γ production (x-axis).

Supplementary Figure 4 Distinct functional subsets of *M. bovis*-specific CD8 T cells following concurrent assessment of proliferation and IFN-γ production. Bar graphs (left) and pie charts (right) showing the frequency of CD4 T cells with distinct functional phenotypes from control (gray bars) and *M. bovis*-infected animals (black bars) at 4**-(A)**, 8**-(B)**, and 48-**(C)** weeks post-infection. Functional phenotypes are denoted as CD8 T cells that in response to *in vitro* PPDb stimulation show proliferation and IFN-γ production (green), IFN-γ production only (blue), proliferation only (purple), or do not respond [double negative (gold)]. Shown are mean frequencies ± S.D.

Supplementary Figure 5 Distinct functional subsets of *M. bovis*-specific γδ T cells following concurrent assessment of proliferation and IFN-γ production. Bar graphs (left) and pie charts (right) showing the frequency of CD8 T cells with distinct functional phenotypes from control (gray bars) and *M. bovis*-infected animals (black bars) at 4**-(A)**, 8**-(B)**, and 48**-(C)** weeks post-infection. Functional phenotypes are denoted as γδ T cells that in response to *in vitro* PPDb stimulation show proliferation and IFN-γ production (green), IFN-γ production only (blue), proliferation only (purple), or do not respond [double negative (gold)]. Shown are mean frequencies ± S.D.

Supplementary Figure 6 Breakdown of *M. bovis-*specific T cell subsets by functional phenotype. Shown are pie charts showing the distribution of CD4 (top row), CD8 (middle row), and γδ (bottom row) T cells responding to antigen stimulation from *M. bovis*-infected animals via proliferation and IFN-γ production (green), IFN-γ production only (blue), and proliferation only (purple).

Supplementary Figure 7 Distinct functional subsets of *M. bovis*-specific CD8 T cells following concurrent assessment of proliferation and IFN-γ production following antigen stimulation and restimulation *in vitro*. Bar graphs (left) and pie charts (right) showing the frequency of CD4 T cells with distinct functional phenotypes from control (gray bars) and *M. bovis*-infected animals (black bars) at 4**-(A)**, 8**-(B)**, and 48**-(C)** weeks post-infection. PBMC were stimulated *in vitro* with PPDb for 7 days and restimulated with PMA/ionomycin overnight for the last 16 h of culture. Proliferation and IFN-γ production were then assessed concurrently via flow cytometry. Functional phenotypes are denoted as CD8 T cells that show proliferation and IFN-γ production (green), IFN-γ production only (blue), proliferation only (purple), or do not respond [double negative (gold)]. Shown are mean frequencies ± S.D.

Supplementary Figure 8 Distinct functional subsets of *M. bovis*-specific γδ T cells following concurrent assessment of proliferation and IFN-γ production following antigen stimulation and restimulation *in vitro*. Bar graphs (left) and pie charts (right) showing the frequency of CD4 T cells with distinct functional phenotypes from control (gray bars) and *M. bovis*-infected animals (black bars) at 4**-(A)**, 8**-(B)**, and 48**-(C)** weeks post-infection. PBMC were stimulated *in vitro* with PPDb for 7 days and restimulated with PMA/ionomycin overnight for the last 16 h of culture. Proliferation and IFN-γ production were then assessed concurrently via flow cytometry. Functional phenotypes are denoted as γδ T cells that show proliferation and IFN-γ production (green), IFN-γ production only (blue), proliferation only (purple), or do not respond [double negative (gold)]. Shown are mean frequencies ± S.D.

Supplementary Figure 9 Breakdown of *M. bovis-*specific T cell subsets by functional phenotype following restimulation. Shown are pie charts showing the distribution of CD4 (top row), CD8 (middle row), and γδ (bottom row) T cells responding to *in vitro* PPDb stimulation from *M. bovis*-infected animals via proliferation and IFN-γ production (green), IFN-γ production only (blue), and proliferation only (purple).
