# Supplementary figures and images for: Enhanced Detection of Mycobacterium bovis-Specific T Cells in Experimentally-Infected Cattle
Source: Front Vet Sci. 2021 Jul 14;8:676710. doi: 10.3389/fvets.2021.676710 (PMC8317970; doi:10.3389/fvets.2021.676710)

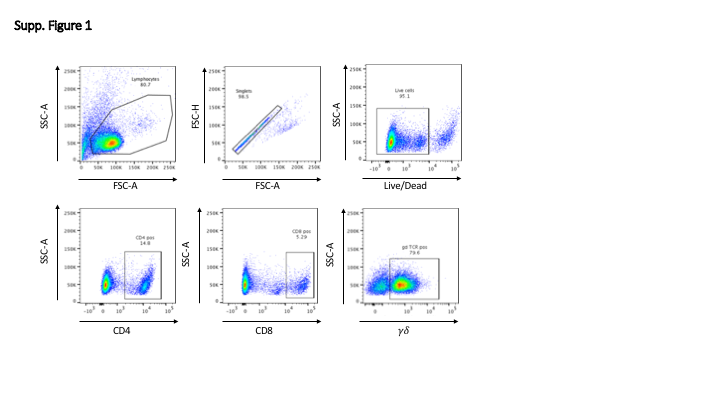

Supplement: Supplementary Figure 1 — Flow cytometry gating scheme for T cell subsets. Shown are representative dot plots demonstrating gating strategies for lymphocytes and singlet discrimination based on FSC and SSC. Also shown are live/dead discrimination based on uptake of a fixable viability dye, and CD4, CD8, and γδ gating based on SSC and fluorescent signal. [file Image_1.TIFF]

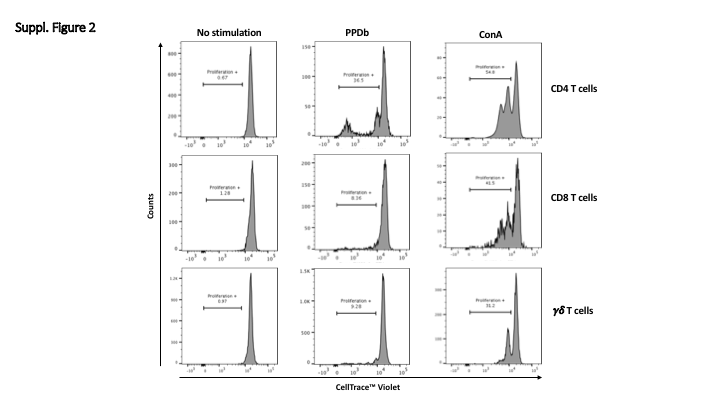

Supplement: Supplementary Figure 2 — Flow cytometry analysis of proliferating cells based on CellTrace™ violet dilution. Shown are representative histograms for determination of the frequency of proliferating CD4, CD8, and γδ T cells without stimulation (first column), PPD-B antigen stimulation (middle column), and ConA stimulation (last column). [file Image_2.TIFF]

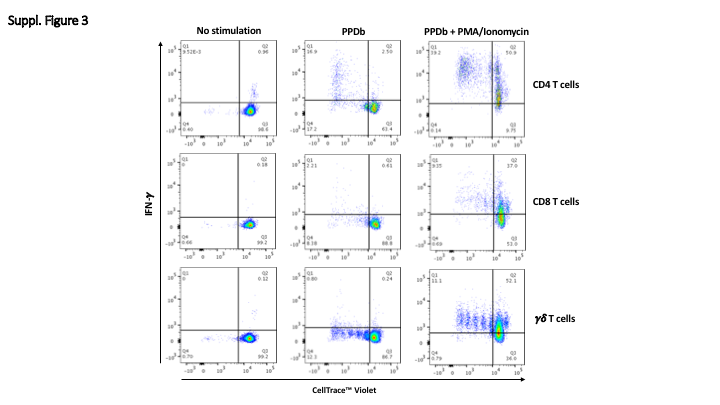

Supplement: Supplementary Figure 3 — Flow cytometry gating for the concurrent assessment of proliferation and IFN-γ responses for T cell subsets. Shown are representative dot plots for cells gated on CD4 (top row), CD8 (middle row), and γδ (bottom row) following in vitro culture without stimulation (first column), with PPDb stimulation (middle column) and PPDb + PMA/Ionomycin (last column), for the assessment of proliferation (CellTrace dilution, y-axis), and IFN-γ production (x-axis). [file Image_3.TIFF]

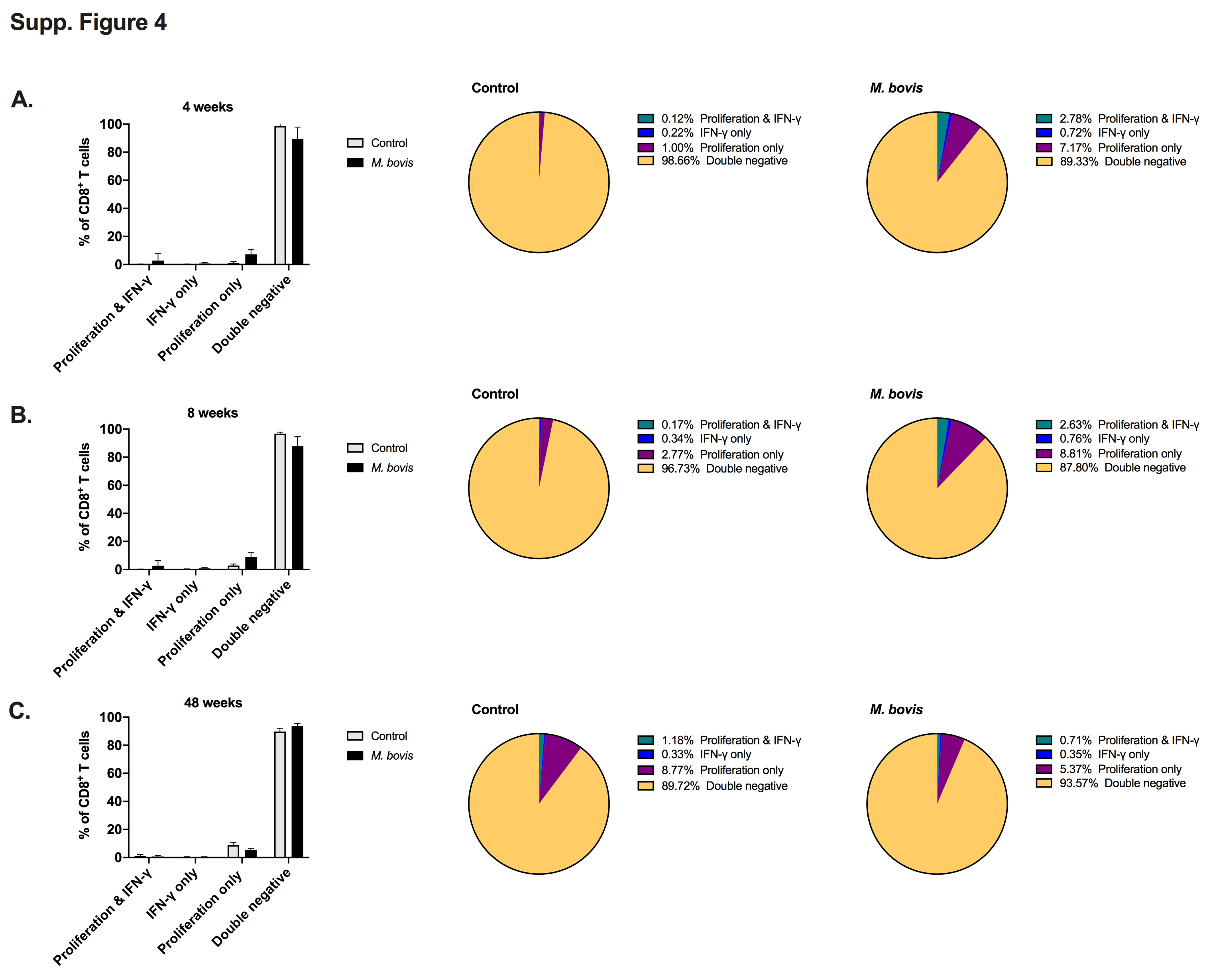

Supplement: Supplementary Figure 4 — Distinct functional subsets of M. bovis-specific CD8 T cells following concurrent assessment of proliferation and IFN-γ production. Bar graphs (left) and pie charts (right) showing the frequency of CD4 T cells with distinct functional phenotypes from control (gray bars) and M. bovis-infected animals (black bars) at 4-(A), 8-(B), and 48-(C) weeks post-infection. Functional phenotypes are denoted as CD8 T cells that in response to in vitro PPDb stimulation show proliferation and IFN-γ production (green), IFN-γ production only (blue), proliferation only (purple), or do not respond [double negative (gold)]. Shown are mean frequencies ± S.D. [file Image_4.TIFF]

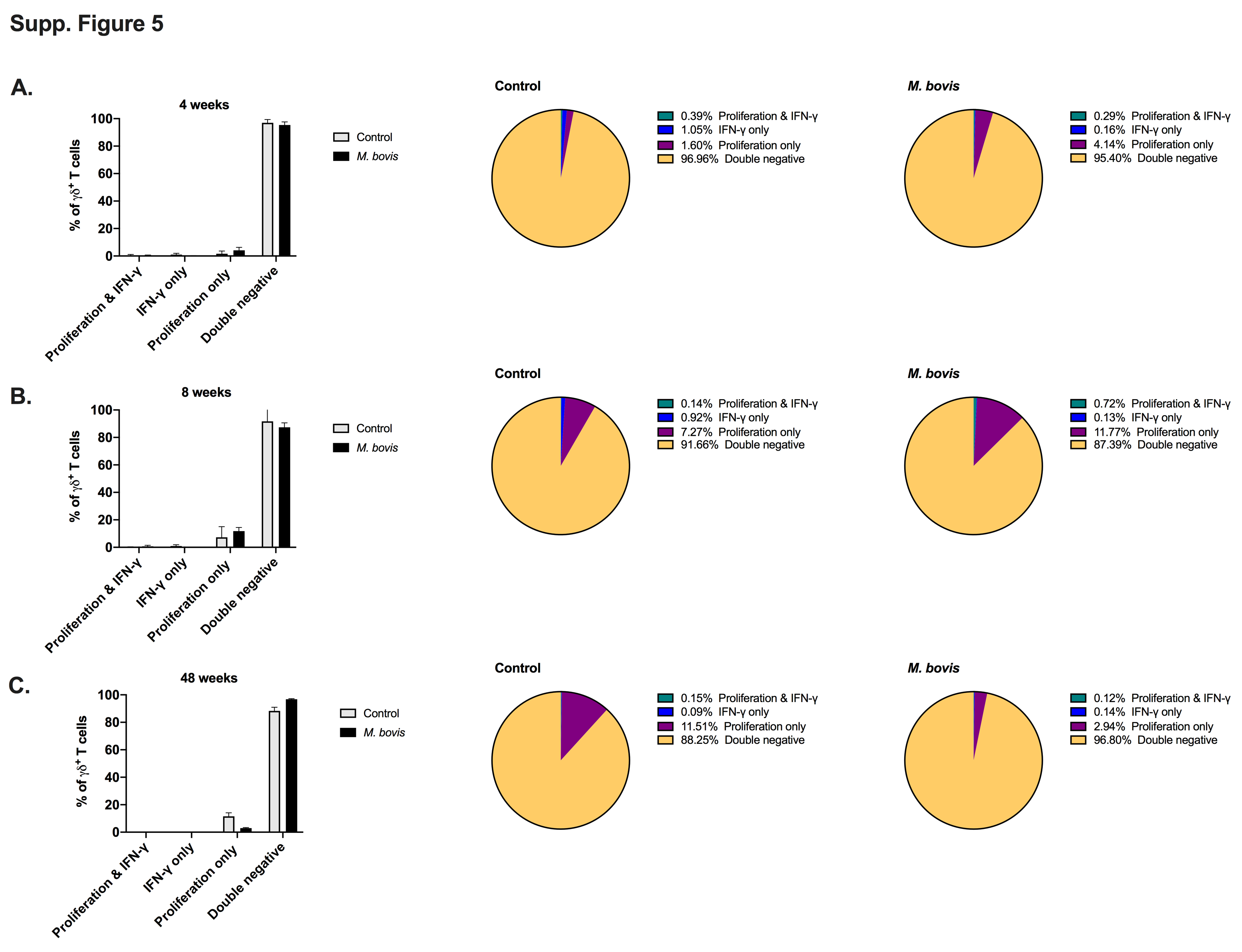

Supplement: Supplementary Figure 5 — Distinct functional subsets of M. bovis-specific γδ T cells following concurrent assessment of proliferation and IFN-γ production. Bar graphs (left) and pie charts (right) showing the frequency of CD8 T cells with distinct functional phenotypes from control (gray bars) and M. bovis-infected animals (black bars) at 4-(A), 8-(B), and 48-(C) weeks post-infection. Functional phenotypes are denoted as γδ T cells that in response to in vitro PPDb stimulation show proliferation and IFN-γ production (green), IFN-γ production only (blue), proliferation only (purple), or do not respond [double negative (gold)]. Shown are mean frequencies ± S.D. [file Image_5.TIFF]

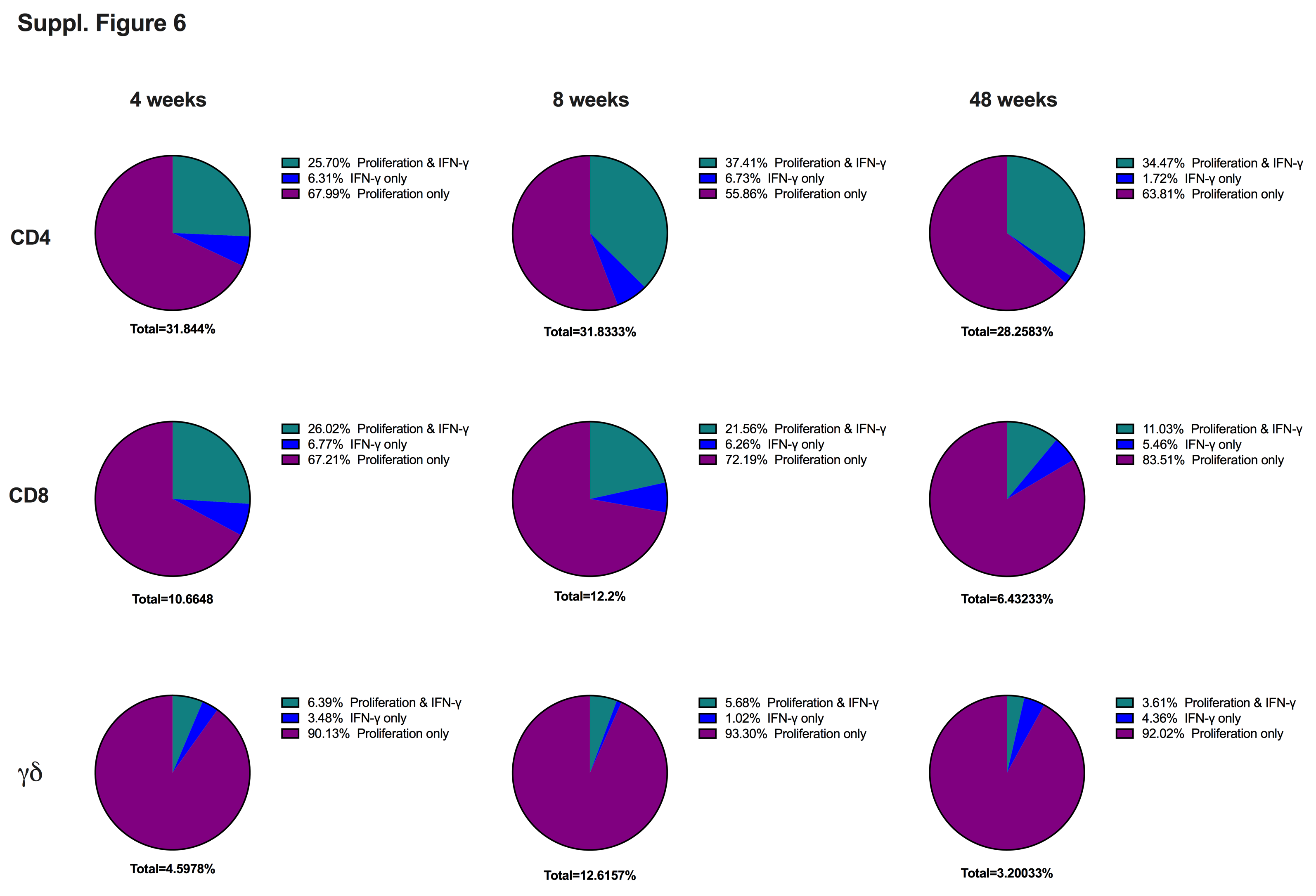

Supplement: Supplementary Figure 6 — Breakdown of M. bovis-specific T cell subsets by functional phenotype. Shown are pie charts showing the distribution of CD4 (top row), CD8 (middle row), and γδ (bottom row) T cells responding to antigen stimulation from M. bovis-infected animals via proliferation and IFN-γ production (green), IFN-γ production only (blue), and proliferation only (purple). [file Image_6.TIFF]

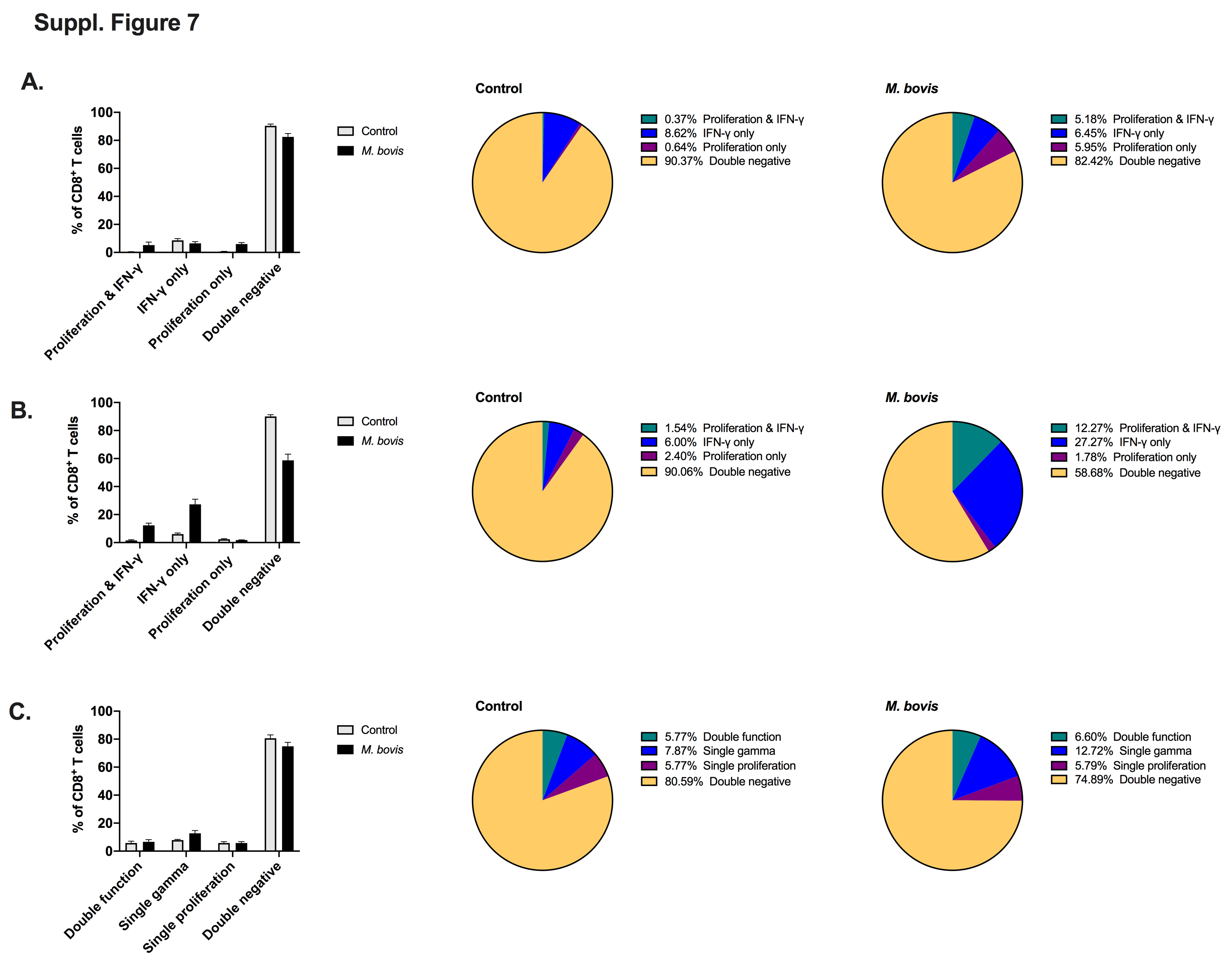

Supplement: Supplementary Figure 7 — Distinct functional subsets of M. bovis-specific CD8 T cells following concurrent assessment of proliferation and IFN-γ production following antigen stimulation and restimulation in vitro. Bar graphs (left) and pie charts (right) showing the frequency of CD4 T cells with distinct functional phenotypes from control (gray bars) and M. bovis-infected animals (black bars) at 4-(A), 8-(B), and 48-(C) weeks post-infection. PBMC were stimulated in vitro with PPDb for 7 days and restimulated with PMA/ionomycin overnight for the last 16 h of culture. Proliferation and IFN-γ production were then assessed concurrently via flow cytometry. Functional phenotypes are denoted as CD8 T cells that show proliferation and IFN-γ production (green), IFN-γ production only (blue), proliferation only (purple), or do not respond [double negative (gold)]. Shown are mean frequencies ± S.D. [file Image_7.TIFF]

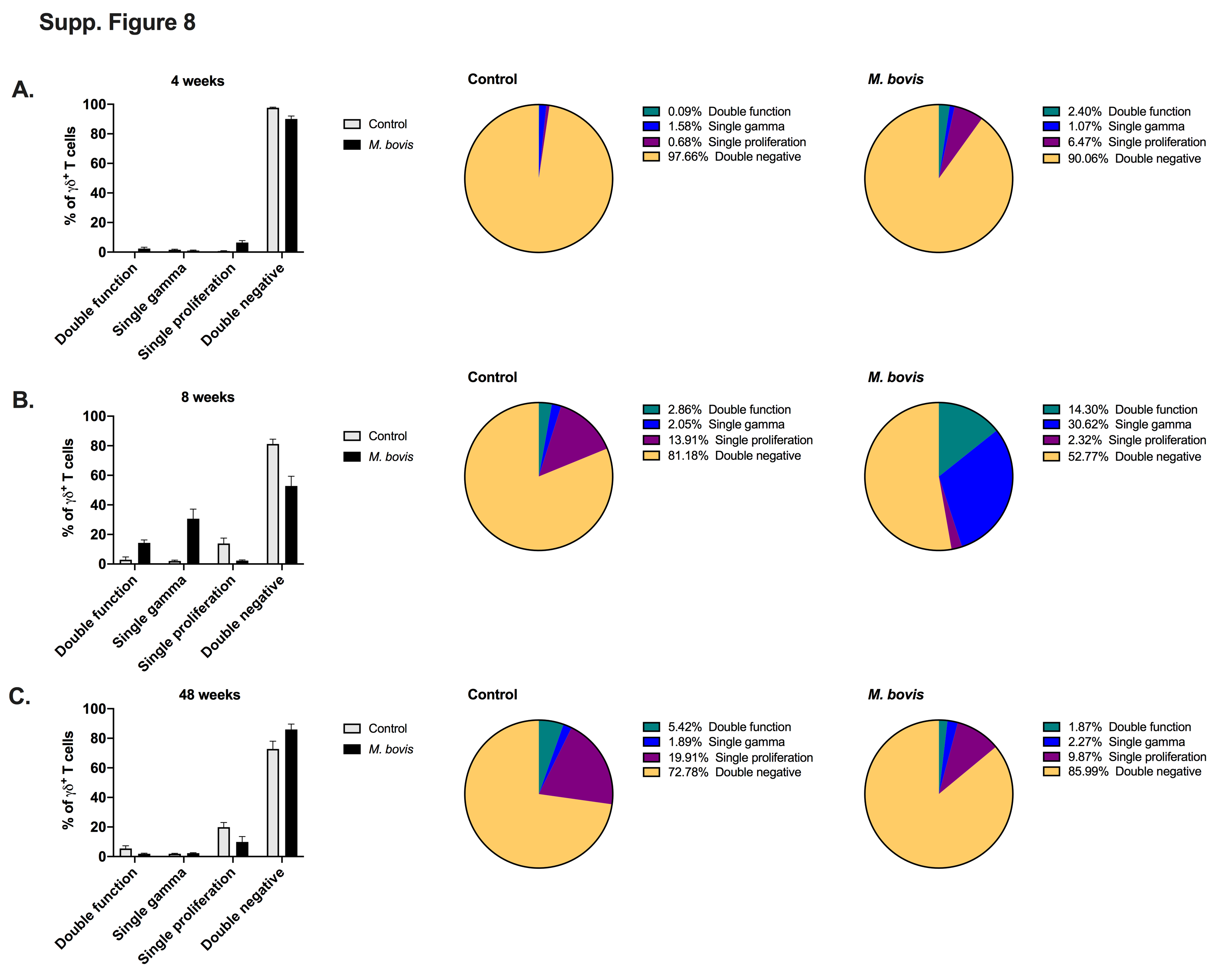

Supplement: Supplementary Figure 8 — Distinct functional subsets of M. bovis-specific γδ T cells following concurrent assessment of proliferation and IFN-γ production following antigen stimulation and restimulation in vitro. Bar graphs (left) and pie charts (right) showing the frequency of CD4 T cells with distinct functional phenotypes from control (gray bars) and M. bovis-infected animals (black bars) at 4-(A), 8-(B), and 48-(C) weeks post-infection. PBMC were stimulated in vitro with PPDb for 7 days and restimulated with PMA/ionomycin overnight for the last 16 h of culture. Proliferation and IFN-γ production were then assessed concurrently via flow cytometry. Functional phenotypes are denoted as γδ T cells that show proliferation and IFN-γ production (green), IFN-γ production only (blue), proliferation only (purple), or do not respond [double negative (gold)]. Shown are mean frequencies ± S.D. [file Image_8.TIFF]

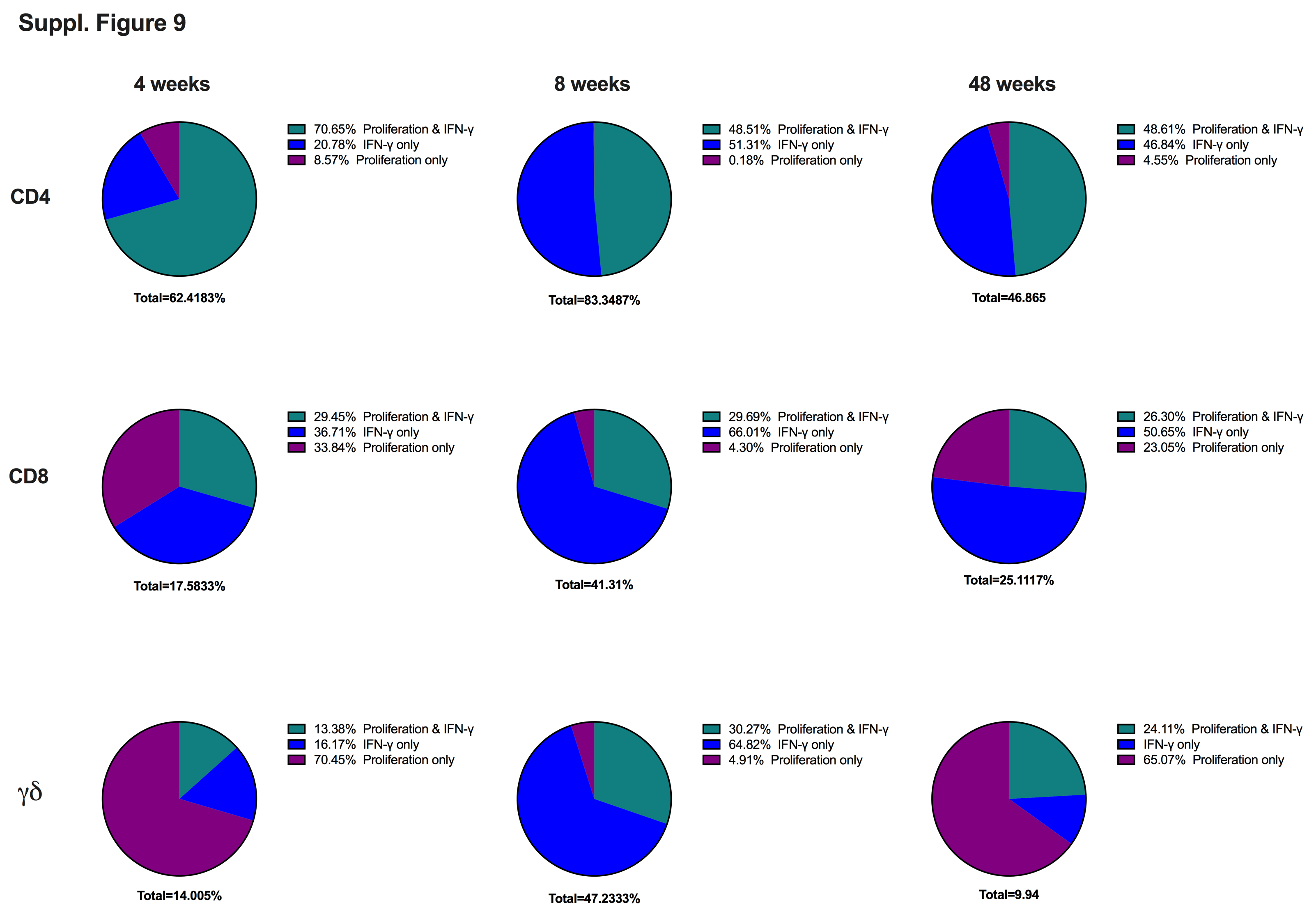

Supplement: Supplementary Figure 9 — Breakdown of M. bovis-specific T cell subsets by functional phenotype following restimulation. Shown are pie charts showing the distribution of CD4 (top row), CD8 (middle row), and γδ (bottom row) T cells responding to in vitro PPDb stimulation from M. bovis-infected animals via proliferation and IFN-γ production (green), IFN-γ production only (blue), and proliferation only (purple). [file Image_9.TIFF]
